# Supplementary material for: Lightning at Jupiter pulsates with a similar rhythm as in-cloud lightning at Earth
Source: Nat Commun. 2023 May 23;14:2707. doi: 10.1038/s41467-023-38351-6 (PMC10205726; doi:10.1038/s41467-023-38351-6)
Supplement: Supplementary file 1 — Supplementary Information [file 41467_2023_38351_MOESM1_ESM.pdf]

Supplementary information

Lightning at Jupiter pulsates with a similar rhythm as in-cloud lightning at Earth

Ivana Kolmašová[1,2*], Ondřej Santolík[1,2], Masafumi Imai[3], William S. Kurth[4], George B. Hospodarsky[4], John E. P. Connerney[5], Scott J. Bolton[6], Radek Lán[1]

[1]Department of Upper Atmosphere, Institute of Atmospheric Physics of the Czech Academy of Sciences, Prague, Czechia

[2]Faculty of Mathematics and Physics, Charles University, Prague, Czechia

[3]Department of Electrical Engineering and Information Science, National Institute of Technology (KOSEN), Niihama College, Niihama, Ehime, Japan

[4]Department of Physics and Astronomy, University of Iowa, Iowa City, Iowa, USA

[5]NASA/Goddard Spaceflight Center, Greenbelt, Maryland, USA

[6]Space Science Department, Southwest Research Institute, San Antonio, Texas, USA

Supplementary Figure 1: Time series of the electric field fluctuations corresponding to time-frequency spectrograms in Fig. 1 in the main paper.

Supplementary Figure 2: Additional examples of groups of JDPs.

Supplementary Figure 3: Distribution of inter-pulse intervals – additional plots.

Supplementary Figure 4: Time series of magnetic field fluctuations

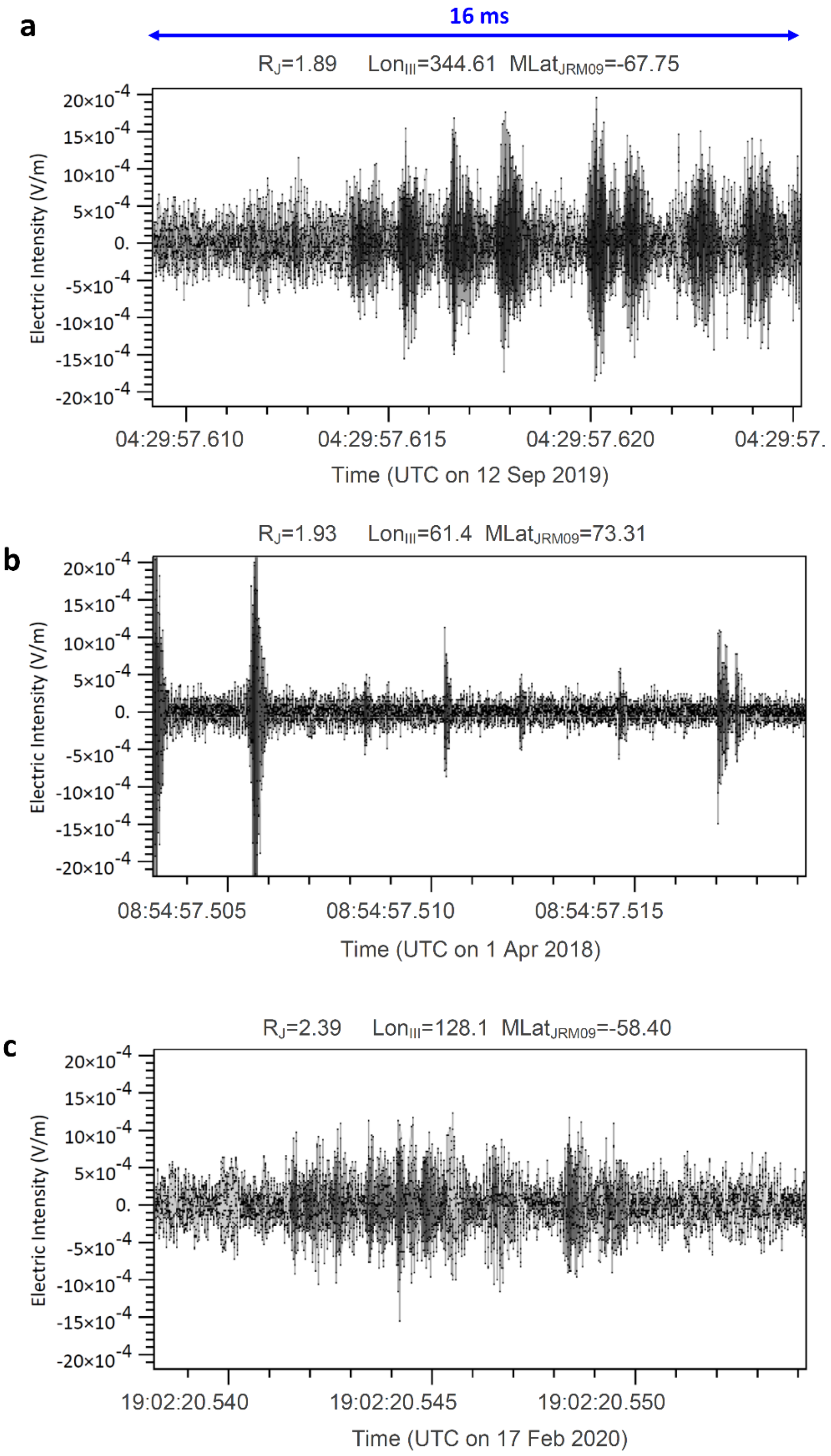

Supplementary Figure 1. Time series of the electric field fluctuations corresponding to time-frequency spectrograms in Fig. 1. a Snapshot recorded on 12 September 2017 after 04:29:57 UTC. **b** Snapshot recorded on 1 April 2018 after 08:54:57 UTC. **c** Snapshot recorded on 17 February 2020 after 19:22:20 UTC.

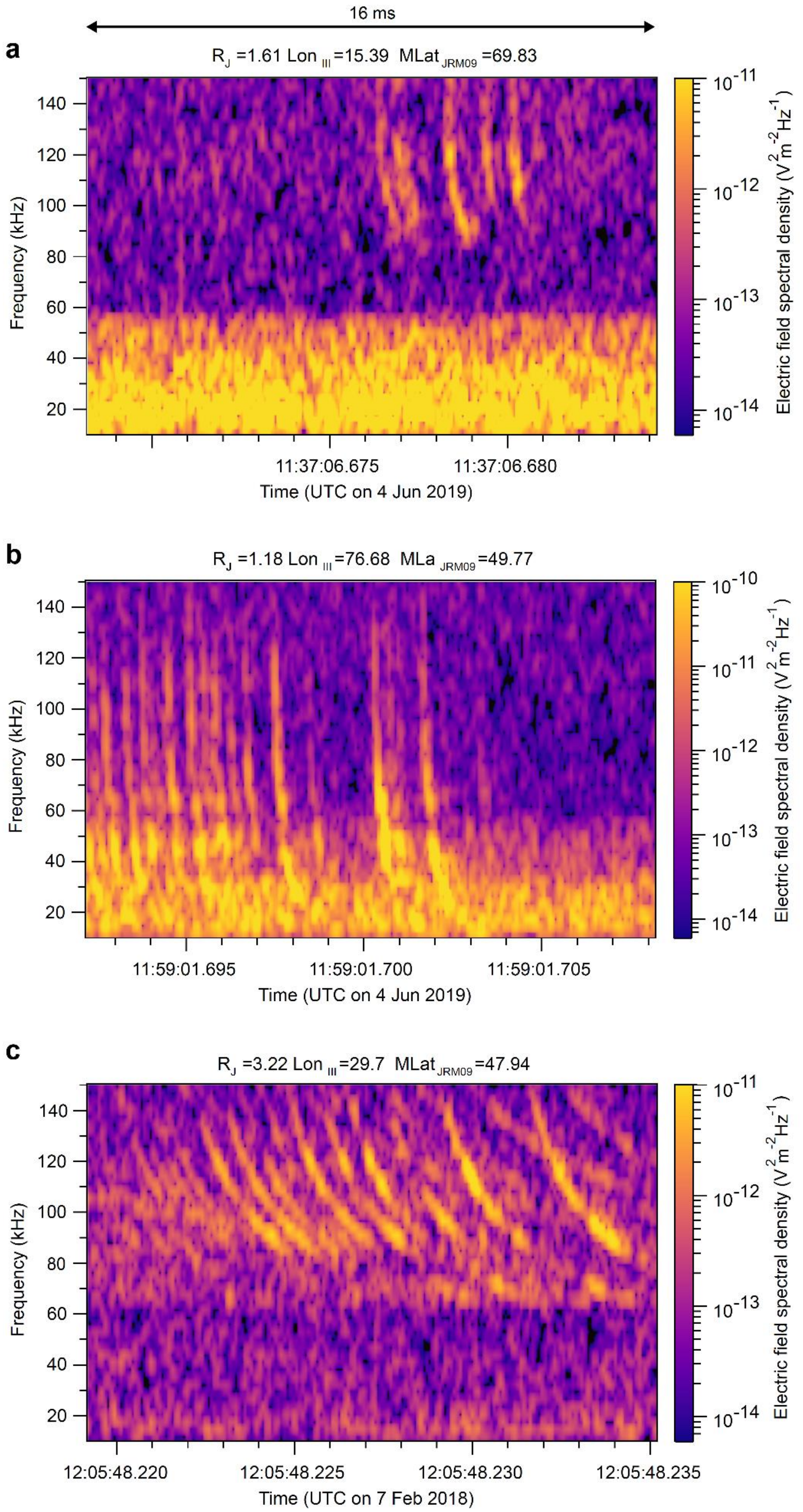
16 ms
a
R_J =1.61 Lon_III =15.39 MLat_JRM09 =69.83
Frequency (kHz)
Electric field spectral density (V^2 m^-2 Hz^-1)
11:37:06.675
11:37:06.680
Time (UTC on 4 Jun 2019)
b
R_J =1.18 Lon_III =76.68 MLa_JRM09 =49.77
11:59:01.695
11:59:01.700
11:59:01.705
Time (UTC on 4 Jun 2019)
c
R_J =3.22 Lon_III =29.7 MLat_JRM09 =47.94
12:05:48.220
12:05:48.225
12:05:48.230
12:05:48.235
Time (UTC on 7 Feb 2018)

Supplementary Figure 2. Additional examples of groups of JDPs. Frequency–time power spectrograms of the electric field fluctuations. **a** Snapshot recorded on 6 June 2019 after 11:37:06 UTC at a distance of 1.61 R_J. **b** Snapshot recorded on 6 June 2019 after 11:59:01 UTC at a distance of 1.18 R_J. **c** Snapshot recorded on 7 February 2018 after 12:05:48 UTC at a distance of 3.22 R_J.

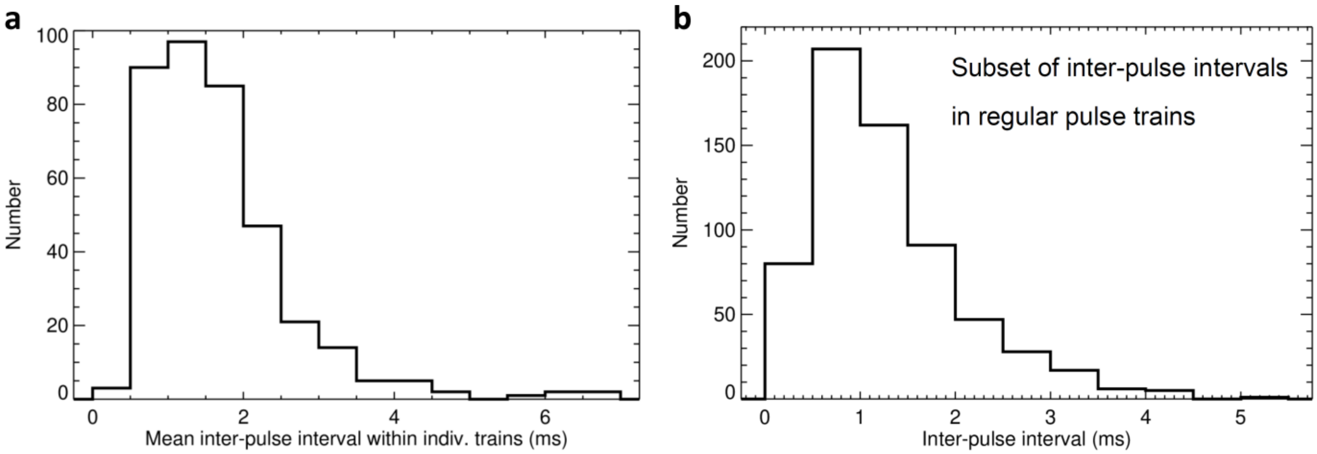

Supplementary Figure 3. Distribution of inter-pulse intervals – additional plots. a Distribution of average inter-pulse intervals calculated separately for each individual group of pulses. **b** Distribution of inter-pulse intervals in regular JDP groups (defined as groups with the standard deviation of their inter-pulse interval smaller than one-half of its mean value). Similar position of the main peak also resulted from preliminary analysis of a smaller subset of JDP intervals[1].

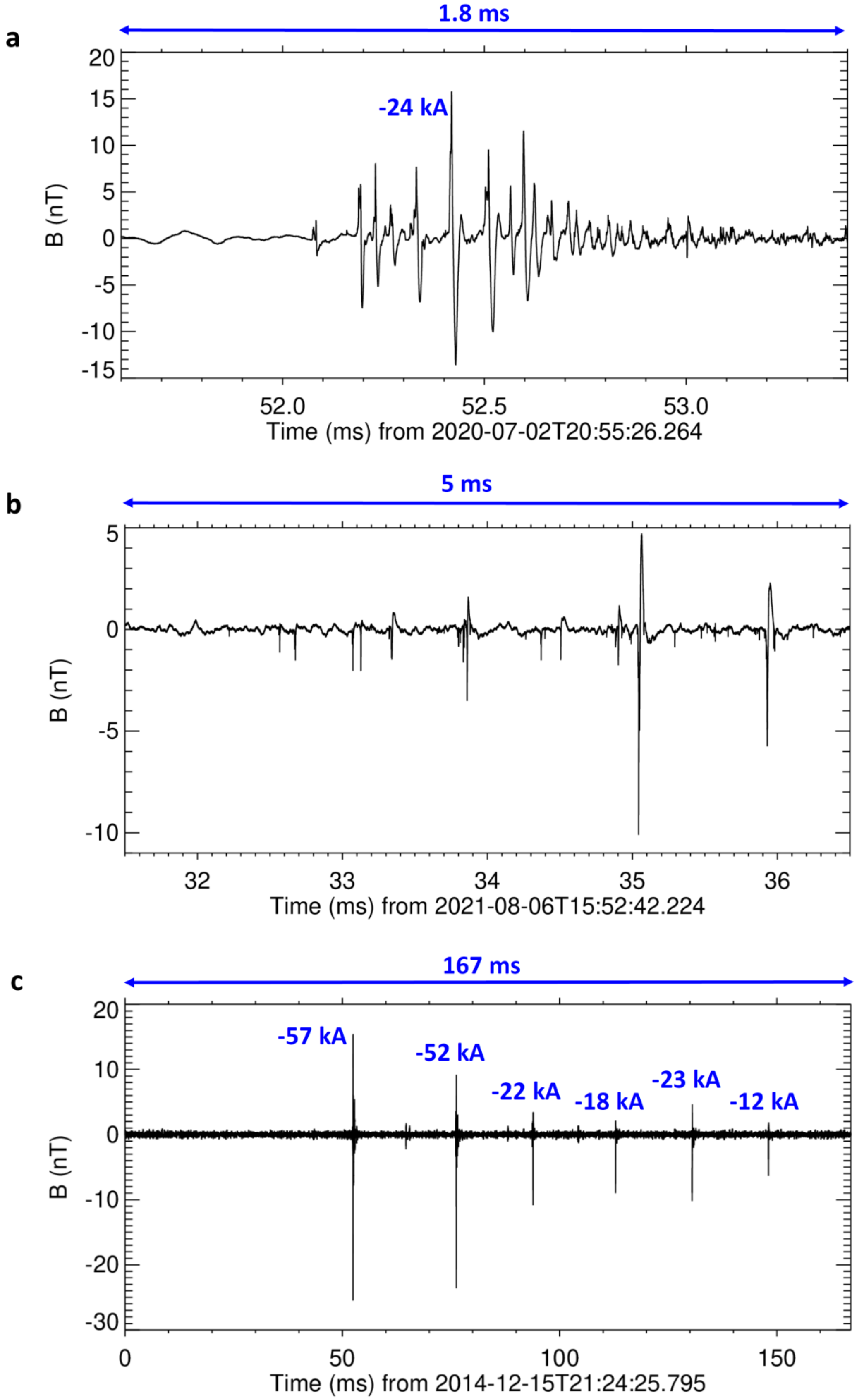
a
1.8 ms
-24 kA
B (nT)
Time (ms) from 2020-07-02T20:55:26.264
b
5 ms
B (nT)
Time (ms) from 2021-08-06T15:52:42.224
c
167 ms
-57 kA
-52 kA
-22 kA
-18 kA
-23 kA
-12 kA
B (nT)
Time (ms) from 2014-12-15T21:24:25.795

Supplementary Figure 4. Time series of magnetic field fluctuations. a 1.8 long ms waveform showing an initiation of an inverted IC flash starting by intense magnetic field IB pulses (-26 kA) occurring on 2 July 2020 at 20:55:26 UTC (detected at the Milešovka observatory, Czechia) - corresponds to the spectrogram in Fig. 4a in the main paper. **b** 5 ms long waveform showing an initiation of a normal IC flash occurring on 6 August 2021 at 15:52:42 UTC (detected at the Dlouhá Louka observatory, Czechia) - corresponds to the spectrogram in Fig.4b in the main paper. **c** 167ms long waveform of a multi-stroke negative cloud-to ground composed of 6 individual strokes (-57 kA, -52 kA, -22 kA, -18 kA, -24 kA, -12 kA) occurring on 15 December 2014 at 21:24:25 UTC in Southern France and detected at the external site of the LSBB laboratory in Rustrel, France. The peak current estimates were provided by the European Lightning location network EUCLID[2].

Suplementary references

1. Imai, M. *et al.* Evidence for low density holes in Jupiter's ionosphere. *Nat. Commun.* (2019) doi:10.1038/s41467-019-10708-w.
2. Poelman, D. , Schultz, W., Pedeboy, S. et al. Global ground strike point characteristics in negative downward lightning flashes – Part 1: Observations. *Nat. Hazards Earth Syst. Sci.* **21**, 1909–1919 (2021).
